# Investigation of the Phenomenological and Psychopathological Features of Trichotillomania in an Italian Sample

**DOI:** 10.3389/fpsyg.2016.00256

**Published:** 2016-02-25

**Authors:** Gioia Bottesi, Silvia Cerea, Enrico Razzetti, Claudio Sica, Randy O. Frost, Marta Ghisi

**Affiliations:** ^1^Department of General Psychology, University of PadovaPadova, Italy; ^2^Department of Health Sciences, University of FirenzeFirenze, Italy; ^3^Smith CollegeNorthampton, MA, USA

**Keywords:** trichotillomania, phenomenology, psychopathology, DSM-5, Italian sample

## Abstract

Trichotillomania (TTM) is still a scarcely known and often inadequately treated disorder in Italian clinical settings, despite growing evidence about its severe and disabling consequences. The current study investigated the phenomenology of TTM in Italian individuals; in addition, we sought to examine patterns of self-esteem, anxiety, depression, and OCD-related symptoms in individuals with TTM compared to healthy participants. The current study represents the first attempt to investigate the phenomenological and psychopathological features of TTM in Italian hair pullers. One hundred and twenty-two individuals with TTM were enrolled: 24 were assessed face-to-face (face-to-face group) and 98 were recruited online (online group). An additional group of 22 face-to-face assessed healthy controls (HC group) was included in the study. The overall female to male ratio was 14:1, which is slightly higher favoring female than findings reported in literature. Main results revealed that a higher percentage of individuals in the online group reported pulling from the pubic region than did face-to-face participants; furthermore, the former engaged in examining the bulb and running the hair across the lips and reported pulling while lying in bed at higher frequencies than the latter. Interestingly, the online TTM group showed greater functional and psychological impairment, as well as more severe psychopathological characteristics (self-esteem, physiological and social anxiety, perfectionism, overestimation of threat, and control of thoughts), than the face-to-face one. Differences between the two TTM groups may be explained by the anonymity nature of the online group, which may have led to successful recruitment of more serious TTM cases, or fostered more open answers to questions. Overall, results revealed that many of the phenomenological features of Italian TTM participants matched those found in U.S. clinical settings, even though some notable differences were observed; therefore, cross-cultural invariance might represent a characteristic of OCD-related disorders.

## Introduction

Trichotillomania (TTM; Hair Pulling Disorder) is a psychological disorder currently included into the Obsessive-Compulsive and Related Disorders category of the Diagnostic and Statistical Manual-Fifth Edition (American Psychiatric Association, 2013). Individuals suffering from TTM are characterized by recurrent hair pulling resulting in hair loss (Criterion A), repeated attempts to decrease or stop the behavior (Criterion B), and clinically significant distress or impairment in social, occupational, or other important areas of functioning due to hair pulling (Criterion C). The hair pulling or hair loss is neither attributable to another medical condition (Criterion D) nor better explained by the symptoms of another mental disorder (Criterion E; American Psychiatric Association, 2013).

Trichotillomania occurs in 1–2% of adolescents and adults (1-year estimated prevalence; American Psychiatric Association, 2013); nonetheless, data regarding prevalence of the disorder are controversial since estimates depend on the restrictiveness of the diagnostic criteria adopted in the different studies (Duke et al., 2010). In clinical settings, women are typically more represented than men (female to male ratio is about 10:1; Keuthen et al., 2001; Miltenberger et al., 2006; Woods et al., 2006a; Lochner et al., 2010; Odlaug et al., 2010; American Psychiatric Association, 2013). The onset of TTM is bimodal with peaks in early childhood and adolescence, but it is most frequently coincident with puberty at about 13 years (Duke et al., 2010; American Psychiatric Association, 2013).

Hair pulling frequency is highly variable. Episodes can be short (lasting a few seconds or minutes) but occur frequently during the day, or they be long (i.e., hours) and occur less frequently (Swedo and Rapoport, 1991). The time spent pulling hair by individuals with TTM ranges between 1 and 3 h a day (O’Sullivan et al., 2000). Individuals with TTM pull out their hair from any region of the body. The most frequently involved areas are the scalp, eyelashes, and eyebrows. Other commonly reported pulling sites are the pubic region, the limbs (Stein and Mansueto, 1999; Wetterneck et al., 2006; Woods et al., 2006a; American Psychiatric Association, 2013), and chest, beard, and mustaches in men (Mansueto et al., 2007; Lochner et al., 2010). Pulling sites often change across time, and people often pull from more than one site. Hair can be pulled by hands or tweezers, by dominant and/or non-dominant hand, in symmetrical ways or not (Walsh and McDougle, 2001), and specific qualities of the hair (e.g., length, color, coarseness) can induce the behavior (O’Sullivan et al., 1997).

Hair pulling usually occurs when no one else is present (Christenson et al., 1991; Casati et al., 2000), and it is frequently endorsed while reading, studying, watching television, driving the car, or lying in bed. Objects, situations, hair qualities, and emotional states have all been suggested as relevant triggers/facilitators of hair pulling (Mansueto et al., 1997). Two TTM subtypes based on pulling style have been identified: automatic and focused hair pulling (Flessner et al., 2008a,b). Automatic hair pulling is characterized by pulling without full behavioral awareness and is generally performed during sedentary situations (e.g., reading or watching television). Focused hair pulling is thought to be affect-driven pulling with full behavioral awareness and triggered by intense emotions or unpleasant internal experiences. Most patients report a mixed pulling style (both automatic and focused; Christenson and Crow, 1996; Flessner et al., 2008a). After pulling, individuals often engage in rituals, such as examining the hair/hair bulb, stroking the hair against the face or tongue, rolling it into a ball, saving it in a special place or manner, running the hair across the lips, biting off the root, mincing or eating the hair or hair bulb (trichophagia; Christenson et al., 1991).

Trichotillomania is associated with a significant impairment. Physical consequences deriving from hair pulling include hair loss, scalp irritation, carpal tunnel syndrome as well as dental and gastrointestinal problems (Bouwer and Stein, 1998; Woods et al., 2001). Psychosocial impairments include difficulty maintaining close relationships and avoidance of social situations and leisure activities (e.g., going to the hair-dresser, to the restaurant, on vacation, etc.; Stemberger et al., 2000; Wetterneck et al., 2006; Woods et al., 2006a). TTM impairs academic functioning by interfering with concentration, studying, and frequently results in missed school days. Individuals with TTM often avoid job interviews or are not able to manage their work duties because of hair pulling (Seedat and Stein, 1998; Keuthen et al., 2002; Woods et al., 2006a). All the above-mentioned difficulties compromise the quality of life of those affected by TTM. In addition, TTM is associated with frequent negative emotional states including sadness, irritability, shame, frustration, guilt, embarrassment, and low self-esteem, as well as the belief that they are unattractive (Casati et al., 2000; Stemberger et al., 2000; du Toit et al., 2001; Diefenbach et al., 2005a,b). Furthermore, individuals with TTM infrequently seek help from mental health professionals (O’Sullivan et al., 1996; Duke et al., 2010), thus resulting in both under-detection and inadequate/absent treatment of the disorder.

TTM is commonly comorbid with obsessive-compulsive disorder (OCD; Swedo and Leonard, 1992; Christenson, 1995), major depressive disorder, anxiety, and substance use disorders (Christenson et al., 1994; Schlosser et al., 1994). Also other body-focused repetitive behaviors such as skin picking and nail biting have been found to co-occur with TTM (Christenson et al., 1991; Stein et al., 2008). Despite evidence concerning comorbid psychopathology in clinical samples, only a few studies have assessed characteristics of individuals suffering from TTM compared to non-clinical controls. Findings from these studies revealed that the former were characterized by higher levels of anxiety and depression (e.g., Bohne et al., 2005, 2008; Diefenbach et al., 2005b; White et al., 2013), and lower self-esteem (e.g., Diefenbach et al., 2005b) than the latter.

Much has been learned about TTM over the last decade. Nonetheless, most literature on clinical samples refers to Caucasian Americans. Few studies have investigated clinical samples of TTM in minority populations or populations outside of North America. It remains to be seen whether TTM symptomatology, phenomenology, and comorbidity are similar across cultures. Neal-Barnett and Stadulis (2006), Neal-Barnett et al. (2010) found that African American and Latino hair pullers showed differences in phenomenology and functional impairment when compared to their Caucasian counterparts, thus highlighting the importance of studying TTM in different cultural contexts. Likewise, very few TTM studies have been reported in European countries. Rufer et al. (2014) examined the association between TTM and alexithymia in a German and Swiss clinical sample. Only one study has examined TTM symptoms in an Italian sample (Ghisi et al., 2013). They reported preliminary data on prevalence, phenomenology, and diagnostic criteria of hair pulling in an Italian non-clinical sample (Ghisi et al., 2013). While the prevalence and phenomenological features were similar to those observed in Caucasian American non-clinical samples, there were some notable exceptions. For instance, legs emerged as a more frequently endorsed pulling site in the Italian than in the U.S. population; moreover, Italian males reported pulling their hair while driving more frequently than did Italian females, whereas the reverse pattern was observed in a U.S. community sample (Duke et al., 2009). To date, however, there have been no studies of clinical samples of TTM patients in Italy.

### The Current Study

This study aimed to examine the phenomenology of TTM in a sample of Italian individuals with TTM. In light of findings by Ghisi et al. (2013), we expected that the phenomenology of TTM would resemble that observed in American TTM patients.

The second objective of the study was to examine emotional characteristics of individuals suffering from TTM in Italy. Specifically, we compared Italian individuals with TTM to healthy participants on measures of self-esteem, anxiety, depression, and OCD-related symptoms. We hypothesized that the former would show lower self-esteem and higher levels of psychopathology (social and physiological anxiety, depression, OCD symptoms) than the latter.

## Materials and Methods

### Participants

One hundred and twenty-two individuals suffering from TTM were enrolled for this study. Twenty-four participants (22 females and 2 males; mean age = 31.08, *SD* = 10.92) responded to advertisements in local newspapers and public settings and came to our laboratory for assessment (face-to-face group). Ninety-eight individuals (92 females and 6 males; mean age = 26.22, *SD* = 7.88) were recruited online and completed an internet-based survey assessing TTM phenomenology (online group). Only a subsample of the online group (*N* = 22) filled-in the whole battery of questionnaires (comprising all measures described in the Measures paragraph). Inclusion criteria for all participants were having endorsed pulling hair (for non-cosmetic purposes) resulting in hair loss at least once a week in the previous 2-weeks period; reporting repeated attempts to decrease or stop the behavior; reporting significant psychological distress due to hair pulling. Exclusion criteria were the existence of severe neurological diseases, current or past psychotic disorders, and mental retardation. An additional group comprising 22 healthy controls (HC group) was included in the study. These were community individuals from different towns in Italy (21 females and 1 male; mean age = 31.38, *SD* = 11.22). To be included in the study, HCs must not report neurological diseases nor meet diagnostic criteria for any psychiatric disorder. Fourteen out of 98 participants included in the online group and 8 out of 24 individuals included in the face-to-face one were medicated [they reported to assume selective serotonin reuptake inhibitors (SSRIs)]. All the HC participants were unmedicated.

In light of the different recruitment strategies employed, a preliminary comparison between the three groups on socio-demographic variables was performed (**Table 1**). No group differences emerged regarding gender, years of education, and occupation. Groups differed in age however. Bonferroni *post hoc* comparisons revealed that individuals in the online group were significantly younger than HCs (*p* = 0.03), but did not differ from those in the face-to-face group (*p* > 0.05). The groups also differed in marital status, since a higher percentage of the online group was single (more than 60%) compared to individuals in the other two groups.

**Table 1:** Socio-demographic variables of the three groups.

	Online group (*N* = 98)	Face-to-face group (*N* = 24)	HC group (*N* = 22)	χ^2^/F	df	*p*
Gender (% females)	93.9	91.7	95.5	0.29	2	0.86
Age	26.22 (7.88)	31.08 (10.92)	31.38 (11.22)	5.14	2,141	0.007
Education (mean years)	14.70 (3.39)	15.20 (3.80)	15.14 (3.80)	0.28	2,141	0.77
Marital status (% single)	63.3	45.8	22.7	40.36	6	<0.001
Occupation (% full-employed)	28.6	16.7	22.7	19.86	14	0.13

### Measures

The *Italian Hair Pulling Questionnaire* (IHPQ; Bottesi et al., in preparation) is a 31-items self-report questionnaire assessing the phenomenology of hair pulling. Since no Italian standardized measures evaluating hair pulling were available, the IHPQ was developed taking into account the most relevant phenomenological characteristics of hair pulling. The IHPQ explores a broad range of features of TTM, such as frequency and duration of episodes; onset and duration of the disorder; members of the family suffering from a similar problem; impairment associated with the behavior; presence of bald spots due to hair pulling and estimated number of hair pulled each episode; primary pulling sites as well as the frequency of the behavior in each site on a five-point Likert scale ranging from 0 = “never” to 4 = “many times per day.” Additional questions concerned hair pulling modalities (e.g., by dominant/non dominant hand; use of tweezers); specific hair qualities triggering the behavior (e.g., texture or color); ritual behaviors endorsed after hair pulling, as well as their frequency (rated on a five-point Likert scale ranging from 0 = “never” to 4 = “always”). Situations in which hair pulling occurred and respective frequency (rated as noted above); feelings, emotions, and sensations experienced before and during hair pulling (rated on a seven-point Likert scale from 0 = “It doesn’t reflect me at all” to 7 = “It reflects me completely”) were examined as well (see **Table 5**, p. 16, for specific items). Psychosocial (e.g., avoidance of activities, social difficulties) and physical impairments due to hair pulling, as well as the presence of past or current help-seeking behaviors and related outcomes were also assessed. Lastly, the questionnaire comprises a section that asks participants to rate the extent to which they had experienced 10 different affective states before, during, and after hair pulling. Findings from these data were part of a separate study and therefore not analyzed in the present paper.

The *Rosenberg Self-Esteem Scale* (RSES; Rosenberg, 1965; Italian version by Prezza et al., 1997) is a 10-items self-report questionnaire measuring global self-esteem. Good internal consistency values have been reported for the original RSES (Gray-Little et al., 1997). The Italian version also showed good psychometric properties (Prezza et al., 1997).

The *Beck Depression Inventory-II* (BDI-II; Beck et al., 1996; Italian version by Ghisi et al., 2006) is a 21-items self-report questionnaire assessing the severity of affective, cognitive, motivational, vegetative, and psychomotor components of depression. The BDI-II showed high internal consistency and good 1-week test–retest reliability among college students (Beck et al., 1996). The Italian version evidenced excellent psychometric properties as well (Sica and Ghisi, 2007).

The *Beck Anxiety Inventory* (BAI; Beck et al., 1988; Italian version by Sica et al., 2006) is a 21-items self-report questionnaire measuring the severity of physiological anxiety. The BAI possesses excellent internal consistency and good 1-week test–retest reliability (Beck et al., 1988). The Italian version of the BAI demonstrated good internal consistency and 30-days test–retest reliability (Sica et al., 2006; Sica and Ghisi, 2007).

The *Social Interaction Anxiety* Scale (SIAS; Mattick and Clarke, 1998; Italian version by Sica et al., 2007) is a 19-items self-report measure designed to assess social interaction anxiety. The SIAS showed strong psychometric properties (Mattick and Clarke, 1998) including high internal consistency and test–retest reliability. The Italian version proved to be highly reliable and stable as well (Sica et al., 2007).

The *Obsessive Compulsive Inventory* (OCI; Foa et al., 1998; Italian version by Sica et al., 2009) is a 42-items self-report questionnaire measuring the frequency and distress caused by OCD symptoms. It is made up of seven subscales: washing, checking, ordering, obsessing, doubting, mental neutralizing, and hoarding. Internal consistency values of the original version were good (Foa et al., 1998) as were those observed in an Italian clinical group (Sica et al., 2009). In the present study only distress associated with obsessions and compulsions has been taken into account since the two scales (frequency and distress) have been demonstrated to yield redundant information (e.g., Foa et al., 2002; Wu and Watson, 2003). The OCI was preferred over the shorter version composed by 18-items (OCI-R) because previous investigations showed that the brevity of the OCI-R scales may be of concern especially for an excessive restriction of score range (Ghisi et al., 2010; Sica et al., 2012).

The *Obsessive Beliefs Questionnaire-87* (OBQ-87; Obsessive Compulsive Cognitions Working Group, 1997, 2001; Italian version by Sica et al., 2004) is an 87-items self-report questionnaire assessing six dysfunctional beliefs identified as central to OCD. The original version of OBQ-87 consists of six subscales that demonstrated good internal consistency values and test–retest reliability in an OCD sample (Obsessive Compulsive Cognitions Working Group, 2001). The Italian version of the questionnaire also consists of six subscales, slightly different from those of the original ones: perfectionism; overestimation of threat; control of thoughts; thought-action fusion; responsibility-omission (an inflated sense of responsibility to ensure that one has prevented a potentially harmful situation); responsibility-harm (an inflated sense of responsibility for actions which are interpreted as negative or immoral; Sica et al., 2004). The Italian version of the OBQ-87 showed good internal consistency for the six scales in an Italian non-clinical sample (Sica et al., 2004).

The *Not Just Right Experiences-Questionnaire Revised* (NJRE-QR; Coles et al., 2003; Italian version by Ghisi et al., 2010) is a self-report measure composed of 19-items. The first 10-items present sample NJREs and respondents have to indicate whether they experienced these within the past month. In the following two-items respondents identify the most recent NJRE and when it last occurred. In the last seven-items, respondents rate frequency, intensity, immediate distress, delayed distress, rumination, urge to respond, and sense of responsibility associated with the most recent NJRE. The sum of ratings for these seven-items comprises the NJRE-QR severity scale. The questionnaire showed good internal consistency and 30-days test–retest reliability, as well as good convergent and divergent validity (Coles et al., 2003). The Italian version of the NJRE-QR demonstrated unidimensionlity and good psychometric properties (Ghisi et al., 2010).

### Procedure

Several recruitment strategies were employed in the present study. Participants in the face-to-face group responded to advertisements in local newspapers and in public settings such as dermatological clinics and beauticians. The announcements contained a brief description of TTM and of the objectives of the current research and invited those interested to contact the last author by phone or email. A psychologist experienced in diagnosing psychiatric disorders, supervised by two Ph.D. level psychotherapists, performed the clinical interview.

To expand the sample size, potential participants were also solicited online. We established a link on the “*trichotillomania global free forum”* (tricotillomania.globalfreeforum.com), a site where people with TTM share opinions and advice. Among the forum’s aims are the dissemination of information about the disorder and the improvement of quality of lives of people suffering from it. Internet sampling procedures have been widely used in TTM research (Woods et al., 2006a; Flessner et al., 2008a,b, 2009; Franklin et al., 2008; Stein et al., 2008; Neal-Barnett et al., 2010; Rufer et al., 2014). The validity of data obtained through web-based surveys has been demonstrated (e.g., Schultz, 1972; Gosling et al., 2004).

Healthy controls were recruited through advertisements in public settings, such as gyms, railway stations, libraries, and university buildings, requesting potential volunteers for psychological studies.

Individuals in the face-to-face and HC groups provided written informed consent before completing study measures. The whole assessment lasted about 1 h. Members of the “*trichotillomania global free forum”* who expressed interest in the study read a description of the study and indicated consent clicking agreement. Subsequently they completed demographic and TTM measures online. Responses were saved anonymously on the Google Drive server and then downloaded for analyses. Those willing to participate further were invited to contact the laboratory providing their e-mail. These individuals completed the additional measures (e.g., RSES; SIAS; BDI-II; BAI; OCI; OBQ-87; NJRE-QR). Only 22 of the 98 online participants completed these measures.

The study was conducted in accordance with the Declaration of Helsinki and approved by the Ethical Committee of the Psychological Sciences, University of Padova. No incentives were offered for participation. The research was funded by the University of Padova (grant CPDR147201/14).

### Statistical Analyses

In order to assess possible differences among groups on socio-demographic variables, chi-squared analyses and one-way analyses of variance (ANOVAs) were conducted. Bonferroni *post hoc* comparisons were conducted to compare the groups when significant differences emerged.

In order to assess the phenomenology of hair pulling behaviors and to verify possible differences between the online and the face-to-face groups in the variables investigated by the IHPQ, descriptive analyses (frequencies and percentages) were performed and chi-squared analyses and ANOVAs were conducted where appropriate.

The online group was significantly younger than the HC group. Consequently, Pearson correlations between age and scores obtained on dependent variables were first performed on the whole sample. Only one significant correlation emerged. Age was negatively correlated with the SIAS (*r* = -0.33, *p* = 0.01). One-way ANOVAs were performed, in order to compare the three groups (online group vs. face-to-face group vs. HC group) on psychological and psychopathological measures. Furthermore, in light of the correlational findings, an analysis of covariance (ANCOVA) was performed on the SIAS controlling for age. Bonferroni *post hoc* comparisons were computed when significant differences emerged.

Conventional significance levels were used (*p* < 0.05). All statistical analyses were conducted using IBM SPSS statistics, version 21.

## Results

### Phenomenological Features of TTM

#### Gender Prevalence, Frequency, and Duration of Episodes, Onset, and Duration of the Disorder

With regard to gender, the overall female to male ratio was 14:1. In the online group the ratio was 15:1, while in the face-to-face group it was 11:1. The difference was not statistically significant ($\chi_{2}^{2}$ = 0.29, *p* = 0.87).

Hair pulling more than once a day was reported by 69.4% of individuals in the online group and 47.8% of those in the face-to-face group. The difference was not statistically significant, however ($\chi_{4}^{2}$ = 8.15, *p* = 0.09). The two groups were also similar as far as the time spent pulling hair each day ($\chi_{4}^{2}$ = 5.47, *p* = 0.24). Just over half (53.3%) spent less than a hour a day, 32.5% spent from 1 to 3 h, 8.3% from 3 to 5 h, 1.7% from 5 to 7 h, and 4.2% more than 8 h. Finally, no difference was observed in the duration of episodes ($\chi_{4}^{2}$ = 4.69, *p* = 0.32). Very few estimated the duration as only 1 min (3.3%), 27.3% estimated the duration between 2 and 5 min, 22.3% between 6 and 10 min, 24.8% between 11 to 30 min, and 22.3% more than 30 min.

The mean age of onset of the disorder was 13.62 years (*SD* = 6.60). Notably, the two groups showed differences in age of onset of the disorder (*F*_1,120_ = 14.92, *p* < 0.001). Those in the online group reported a significantly earlier onset (*M* = 12.54, *SD* = 5.56) than the face-to-face one (*M* = 18.04, *SD* = 8.57). On the other hand, no differences between groups in the duration of the disorder were observed (*F*_1,120_ = 0.00, *p* < 0.99; *M_total_* = 13.26, *SD_total_* = 8.48). No difference between groups was observed on the frequency of one or more members of their family suffered from a similar problem. For the combined TTM groups, 14.8% reported one or more family members with similar problems.

#### Phenomenology of Hair Pulling

Nearly 80% (78.3%; 94 out of 122) of participants indicated the presence of bald spots due to hair pulling. For about a third (32.6%) the bald spot was less than 1 cm in diameter, for 44.2% the diameter of bald area ranged between 1 and 5 cm, for 17.9% it was between 6 to 10 cm and the remaining 5.3% as more than 10 cm. Regarding the number of hairs pulled during each episode, 8.2% estimated pulling only one hair per episode, 26.2% estimated pulling 2–5 hairs, 23% pulled 6–10 hairs, 29.5% pulled 11–30 hairs, and 13.1% more than 30 hairs. The online and the face-to-face groups did not differ in the size of bald spots or number of hairs pulled per episode (all *p*s > 0.05).

**Table 2** shows the percentages of participants who reported a frequency of hair pulling ranging from “once a week” to “several times a day” in relation to different pulling sites. The most frequently involved bodily areas were the scalp, pubic region, and eyebrows. No difference between groups in favorite pulling sites emerged except for the pubic region. A higher percentage of individuals in the online group reported pulling from the pubic region than did face-to-face participants.

**Table 2:** Percentages of participants who reported a frequency of hair pulling ranging from “once a week” to “several times a day” in relation to different pulling sites.

	Total sample (*N* = 122)	Online group (*N* = 98)	Face-to-face group (*N* = 24)		
Body area	%	%	%	χ^2^_(4)_	*p*
Scalp	80.3	82.7	70.8	5.39	0.25
Pubic region	35.2	41.8	8.3	9.62	0.04
Eyebrows	34.4	34.7	33.3	3.84	0.43
Eyelashes	21.3	23.5	12.5	5.52	0.24
Legs	17.2	18.4	12.5	1.94	0.75
Armpit	10.7	12.2	4.2	1.89	0.60
Mustaches	5.7	7.1	5.7	1.82	0.77
Beard	5.7	6.1	4.2	2.40	0.66
Arms	5.7	5.1	8.3	4.35	0.23
Face	9	11.2	0	2.96	0.56
Stomach	3.3	4.1	0	1.01	0.60
Chest	2.5	3.1	0	0.75	0.69
Back	0	0	0	–	–

Over half of the whole TTM sample reported pulling with their dominant hand (59.5%), 37.7% reported pulling with their non-dominant hand, and the remaining by both hands. Pulling by tweezers was reported by 15.6% of participants. Regarding urges, 64.8% reported the need to pull the hair bulb, while 57.4% pulled the hair only with certain fingers. Regarding hair qualities, 62.3% of participants reported that they were driven to pull coarse hair, while 34.4% pulled hair perceived as irregular, 30.3% pulled hair perceived to be shorter than others, 27% pulled hair with split ends, and 26.2% pulled hair that did not “feel right.” No difference between groups emerged in these pulling experiences (all *p*s > 0.05).

Rituals endorsed by participants after hair pulling are shown in **Table 3**. The most frequent rituals were dropping the hair on the floor and examining the hair bulb. Differences between groups emerged in the percentages of individuals engaging in examining the bulb and running the hair across the lips. Those in the online group engaged in these behaviors at higher frequencies than those in the face-to-face group. Biting, chewing or mincing the hair with teeth after pulling them was observed in 29.5% of participants. Nearly a quarter reported eating or swallowing the hair. No difference between groups in these oral behaviors was observed. About half (51.6%) of participants reported that hair pulling occurred both when they were alone and in presence of other people, whereas 47.5% reported pulling hair exclusively when alone and 0.9% exclusively in presence of other people. Hair pulling most often occurred while engaged in other activities such as watching television, reading, studying, or speaking on the phone. However, a substantial percentage reported pulling while lying in bed. Percentages of participants who reported a frequency of hair pulling ranging from “seldom” to “always” in relation to different environmental cues are reported in **Table 4**. Only one difference between groups emerged. Individuals in the online group reported pulling while lying in bed more frequently than those in the face-to-face group.

**Table 3:** Percentages of participants who reported a frequency of hair pulling ranging from “seldom” to “always” in relation to different behaviors that occur after hair pulling.

	Total sample (*N* = 122)	Online group (*N* = 98)	Face-to-face group (*N* = 24)		
Behaviors after pulling	%	%	%	χ^2^_(4)_	*p*
Drop the hair on the floor	77.9	79.6	70.8	3.61	0.46
Examine the bulb	75.4	79.6	58.3	11.49	0.02
Roll the hair between the fingers	68	72.4	50	6.03	0.20
Look the fallen hair on the floor	56.6	57.1	54.2	1.69	0.79
Run the hair between lips	56.6	64.3	25	14.98	0.005
Twist hair with fingers	51.6	50	58.3	1.53	0.82
Touch or rub the hair on face	41	46.9	16.7	8.97	0.06
Bite the bulb	37.7	40.8	25	4.34	0.36
Bite or chew the hair	29.5	33.7	12.5	5.36	0.25
Mince the hair with teeth	29.5	64.7	8.3	9.35	0.05
Saving the hair	25.4	28.6	12.5	4.89	0.30
Eat or swallow the hair	24.8	27.8	12.5	5.03	0.28
Stick the hair on the mirror	20.5	24.5	4.2	5.16	0.27
Sniff the hair after pulling	9.8	10.2	8.3	2.18	0.70

**Table 4:** Percentages of participants who reported a frequency of hair pulling ranging from “seldom” to “always” in relation to different environmental cues.

	Total sample (*N* = 122)	Online group (*N* = 98)	Face-to-face group (*N* = 24)		
Situations in which hair-pulling happens	%	%	%	χ^2^_(4)_	*p*
Watching television	88.5	96.8	79.2	3.54	0.47
Reading	84.4	86.7	75	5.71	0.22
Studying	81.1	82.7	75	7.02	0.13
Speaking on the phone	77.9	80.6	66.7	4.51	0.34
Lying in bed	73.8	80.6	45.8	18.29	0.001
In the bathroom	54.9	38.8	29.2	0.88	0.93
Working	49.2	53.1	33.3	3.79	0.43
Looking in the mirror	45.9	50	29.2	5.16	0.27
In the classroom	44.3	44.9	41.7	7.51	0.11
Driving	36.9	61.2	29.2	8.69	0.07
Speaking in public	30.3	30.6	29.2	2.63	0.45

A comparison between groups regarding the feelings, emotions, and sensations experienced before and during hair pulling indicated that the online group pulled hair more often when feeling an itch than did the face-to-face group (**Table 5**). Furthermore, those in the online group reported more often feeling like they were in trance and losing control when pulling hair than those in the face-to-face group. On the contrary, individuals in the face-to-face group reported more often pulling their hair when facing an unpleasant situation than did their online counterparts.

**Table 5:** Comparison between the two differently recruited groups on TTM phenomenological features.

	Online group (*N* = 98)	Face-to-face group (*N* = 24)	*F*_1,120_	*p*
I pull my hair to get rid of unpleasant emotions	4.45 (2.15)	3.87 (2.77)	1.24	0.27
I pull my hair to get rid of unpleasant thoughts	4.32 (2.15)	3.64 (2.80)	1.60	0.21
I pull my hair when I have to face an unpleasant situation	1.60 (2.30)	4.56 (2.68)	29.09	<0.001
I pull my hair when I’ve itch	4.79 (2.15)	0.35 (0.77)	94.70	<0.001
I start to pull my hair intentionally	2.67 (2.54)	2.96 (2.62)	0.23	0.63
Generally I don’t realize that I’m pulling my hair until I stop	3.31 (2.60)	2.96 (2.75)	0.33	0.57
I don’t realize to pull my hair while I’m pulling	3.02 (2.33)	3.48 (2.89)	0.65	0.42
I pull my hair when I’m focused on doing another activity	4.49 (2.54)	4.78 (2.70)	0.24	0.62
I pull my hair when I think something that is not associated with pull	3.87 (2.77)	3.87 (2.90)	0.00	0.99
When I pull my hair I feel like I’m in trance	5.14 (2.41)	2.26 (2.94)	24.47	<0.001
When I pull my hair I have the feeling of losing control	4.68 (2.53)	2.26 (2.77)	16.44	<0.001

Differences emerged in the amount of distress caused by hair pulling as 73.4% of individuals in the online group reported to be “much” or “very much” distressed, whereas only 30.4% of those in the face-to-face group reported such high levels of distress ($\chi_{4}^{2}$ = 31.08, *p* < 0.001). Furthermore, 74.5% of online participants reported experiencing the impulse to pull “very often” or “almost constantly,” whereas only 33.3% of the face-to-face group did so ($\chi_{4}^{2}$ = 22.50, *p* < 0.001). Overall, 33.6% of participants had sought help previously from a health professional (in particular, psychologists, psychiatrists, or dermatologists), while only the 22.7% of them experienced improvements after these interventions. No difference between groups emerged for these variables (all *p*s > 0.05).

#### Psychosocial and Physical Impairment

Regarding psychosocial impairment, 39.3% of participants reported avoiding leisure activities (e.g., going to the hair-dresser, practicing sports, going to the swimming pool) and 22.1% reported avoiding social events because of the disorder; 32% reported problems in studying or working, 29% had difficulties in relationships with their relatives, and 27% had difficulties in relationships with classmates or colleagues. The online group reported higher levels of impairment than face-to-face participants in the following areas: leisure activities, social events, relationships with relatives, studying/working, and relationships with classmates/colleagues (all *p*s < 0.05). Regarding physical impairments, 7.1% of individuals in the online group reported problems such as dermatitis and skin diseases, whereas no one of those in the face-to-face group did. However, this difference was not statistically significant ($\chi_{1}^{2}$ = 1.82, *p* = 0.18; **Table 6**).

**Table 6:** Percentages of participants who reported psychosocial or physical impairment.

	Total sample (*N* = 121)	Online group (*N* = 98)	Face-to-face group (*N* = 24)		
Situations in which hair-pulling happens	%	%	%	χ^2^_(1)_	*p*
Leisure activities	39.3	44.9	16.7	6.44	0.01
Studying/working	32	36.7	12.5	5.21	0.02
Relationships with classmates/colleagues	27	31.6	8.3	5.30	0.02
Social events	22.1	26.5	4.2	5.59	0.02
Relationships with relatives	18.9	22.4	4.2	4.21	0.04
Physical impairments	5.7	7.1	0	1.82	0.18

### Differences between Groups on Measures of Psychopathology

The groups differed on a number of measures of psychopathology. Means, standard deviations and comparisons are reported in **Table 7**. The online group reported significantly lower self-esteem (RSES) than the face-to-face group (*p* = 0.04) and the HC group (*p* < 0.001), which did not differ from each other (*p* = 0.23).

**Table 7:** Comparison between the TTM groups and healthy participants on psychological and psychopathological features.

	Online subgroup (*N* = 22)	Face-to-face group (*N* = 24)	HC group (*N* = 22)	*F*_2,65_	*p*	η^2^
RSES	26.09 (4.78)	29.83 (6.03)	32.50 (3.78)	9.23	<0.001	0.22
SIAS	34.82 (9.94)	21.45 (13.95)	17.86 (8.66)	13.84	<0.001	0.31
OCI-washing	5.14 (4.52)	4.61 (5.39)	3.09 (3.11)	1.25	0.29	0.04
OCI-checking	7.83 (6.54)	5.52 (5.33)	2.36 (1.62)	6.46	0.003	0.18
OCI-doubting	4.14 (4.10)	2.48 (2.59)	0.50 (0.91)	9.01	<0.001	0.22
OCI-ordering	5.59 (4.88)	3.09 (3.23)	2.27 (2.41)	4.95	0.01	0.13
OCI-obsessing	10.18 (7.82)	7.65 (6.48)	2.73 (3.59)	8.16	0.001	0.20
OCI-hoarding	4.59 (3.42)	3.17 (3.39)	2.04 (1.76)	4.06	0.02	0.11
OCI-neutralizing	4.54 (2.67)	3.56 (3.65)	1.64 (2.17)	5.69	0.005	0.15
BAI	16.05 (9.34)	12.79 (6.86)	8.41 (6.22)	5.34	0.007	0.15
BDI-II	18.23 (8.20)	12.87 (7.39)	5.04 (3.05)	22.03	<0.001	0.41
OBQ-perfectionism	57.75 (22.31)	41.42 (18.29)	31.00 (12.17)	11.78	<0.001	0.27
OBQ-responsibility for harm	60.38 (14.52)	51.96 (17.63)	49.68 (10.26)	3.23	0.05	0.09
OBQ-overestimation of threats	38.74 (14.42)	29.46 (14.04)	24.41 (7.25)	7.04	0.002	0.19
OBQ-control of thoughts	51.90 (17.25)	39.54 (20.02)	31.77 (14.13)	7.30	0.001	0.19
OBQ-responsibility by omission	16.14 (8.22)	12.92 (8.34)	10.68 (4.02)	3.14	0.05	0.09
OBQ-TAF	15.68 (8.53)	12.75 (7.29)	12.95 (4.78)	1.19	0.31	0.04
NJRE-QR	29.50 (12.44)	23.50 (11.44)	13.41 (6.71)	13.13	<0.001	0.29

Both the online and the face-to-face groups scored higher than the HC group on the BDI-II (*p* < 0.001 and *p* = 0.001, respectively), while the online group scored higher than the face-to-face group (*p* = 0.03). On the BAI, the online group scored higher than HC group (*p* = 0.01), but the face-to-face group did not differ from either online (*p* = 0.49) or HC group (*p* = 0.15). The ANCOVA on the SIAS indicated that the online group was more socially anxious than the face-to-face (*p* < 0.001) and the HC (*p* < 0.001) groups, whereas no difference between the face-to-face group and HC group (*p* = 0.99) emerged.

With respect to OCD symptoms, the ANOVAs revealed differences among the groups on all the OCI subscales with the exception of washing. The online group scored significantly higher on checking, doubting, ordering, hoarding, and neutralizing than the HC group (*ps* < 0.05), while the face-to-face group did not differ from either the online or the HC groups (*p*s > 0.05). Both the online and the face-to-face groups scored higher than the HC group on the obsessing subscale (*p* = 0.001 and *p* = 0.03, respectively), whereas no difference between the two TTM samples emerged (*p* = 0.53).

Significant differences between the groups were found for the OBQ-87 perfectionism, overestimation of threat, and control of thoughts. In each case, the online group had higher scores than the face-to-face and HC groups (*ps* < 0.05), while no difference emerged between the face-to-face and HC groups (*ps* > 0.05). There was no difference among the groups on the responsibility for harm, responsibility by omission, or TAF subscales of the OBQ-87 (all *p*s ≥ 0.05).

Finally, significant differences among the groups emerged on NJRE-QR severity scale. Both the online group and the face-to-face groups scored higher than HC group (*p* < 0.001 and *p* = 0.01, respectively), while the online and face-to-face groups did not differ from each other (*p* = 0.17).

## Discussion

Trichotillomania is still scarcely known and often inadequately treated in Italian clinical settings, despite growing evidence about the severe and disabling consequences of this disorder (e.g., Wetterneck et al., 2006; Woods et al., 2006a; American Psychiatric Association, 2013). This study represents the first attempt to characterize the phenomenology and associated psychopathology of people suffering from TTM in Italy.

Many of the characteristics of Italian TTM participants matched those found in U.S. clinical settings. Similar to findings in the U.S., women predominate in this sample. However, the gender ratio was slightly higher favoring females (14:1) than in U.S. samples (10:1; Keuthen et al., 2001; Miltenberger et al., 2006; Woods et al., 2006a; Lochner et al., 2010; Odlaug et al., 2010; American Psychiatric Association, 2013). The average age of onset of the disorder was just over 13 years-old, mirroring U.S. samples (Duke et al., 2010; American Psychiatric Association, 2013). In the current study, as well as U.S. studies, hair pulling frequency and the duration of episodes was highly variable (Swedo and Rapoport, 1991). Pulling sites in the Italian sample were also consistent with findings from other studies (Wetterneck et al., 2006; Woods et al., 2006a) with the scalp, eyebrows, and pubic region being the most frequently involved. Pulling behaviors such as use of the dominant hand and pulling with certain fingers were common. Triggering phenomena such as hair characteristics (e.g., texture, irregularity, etc.) and “not just right” feelings were common in Italian TTM participants, as they have been in U.S. studies (Mansueto et al., 1997; O’Sullivan et al., 1997). Post-pulling behaviors (dropping hair, examining bulb, biting, etc.) were similar to those seen in U.S. samples (Christenson et al., 1991; Schlosser et al., 1994; Grant and Odlaug, 2008). Finally, like U.S. TTM patients (Stemberger et al., 2000; Wetterneck et al., 2006; Woods et al., 2006a; Flessner et al., 2008a), Italian TTM participants suffered considerable distress and impairment.

There were several notable differences between the Italian TTM participants and U.S. TTM patients. More than half of the Italian TTM participants reported spending less than an hour a day pulling their hair, while existing U.S. studies suggest those suffering from TTM typically spend between 1 and 3 h a day pulling (O’Sullivan et al., 2000). The rate of trichophagia (eating hair) was slightly higher in Italian TTM participants than rates reported in U.S. clinical samples (Christenson et al., 1991; Schlosser et al., 1994; Grant and Odlaug, 2008). While studies using U.S. samples suggest that the majority of pulling occurs when alone (Christenson et al., 1991; Casati et al., 2000), more than half of Italian TTM participants reported pulling both when alone and in the presence of others. This contrasts somewhat with findings from an Italian community sample (Ghisi et al., 2013) that the majority (60.5%) pulled hair when alone.

The findings from the present study raise questions about the impact of different recruitment and administration strategies in TTM research. There were notable differences between the two TTM groups in the study. With respect to hair pulling, the online group had an earlier age of onset, and was more likely to pull hair from the pubic region, a finding also reported by Wetterneck et al. (2006). Online participants reported more post-pulling examination of the hair bulb and running the hair across the lips. They reported a higher frequency of pulling when lying in bed (e.g., not engaged in another behavior). Regarding the experiences during pulling, those in the online group reported pulling when feeling an itch, and were more likely to report feeling like they were in a trance or losing control when pulling. The face-to-face group reported pulling in response to an unpleasant situation more than the online participants. Some investigators have characterized TTM patients in terms of pulling styles characterized by whether the behavior is focused or automatic (Flessner et al., 2008a,b). In the present study, the online group displayed more automatic hair pulling features, whereas the face-to-face group was more prone to engage in focused hair pulling. Except from the study by Wetterneck et al. (2006), to our knowledge no previous research directly compared two differently recruited samples of individuals reporting hair pulling behaviors. Notably, online TTM sufferers reported more functional impairment than face-to-face ones. Indeed, they reported higher levels of distress, avoiding more leisure activities and social events, and having more difficulties in sentimental relationships, studying/working, and relationships with classmates/colleagues than their face-to-face counterparts. Furthermore, the former also reported physical impairments (i.e., clinically significant dermatitis and skin diseases) due to the disorder, while the latter did not.

Moreover, those in the online group were more psychologically impaired than their face-to-face counterparts. They had lower self-esteem, higher social anxiety, and higher levels of perfectionism than individuals in the face-to-face and in the HC groups, which did not differ each other. Furthermore, hair pullers in the online group showed higher levels of physiological anxiety, OCD symptoms (such as checking, doubting, ordering, hoarding, and mental neutralizing), overestimation of threat and overimportance attributed to the control of thoughts than the HCs, whereas the hair pullers in the face-to-face group did not differ from the other two groups. Online participants reported more depression than face-to-face participants.

Differences between the two TTM groups may be a function of the anonymity characteristic of the online group. It may have led to successful recruitment of more serious TTM cases, or prompted more honest and open answers to questions. For instance, reporting of hair pulling from pubic areas may be under-reported in face-to-face interviews. In either case, it suggests that data collected from face-to-face interviews may not adequately represent the severity of the disorder. A more controlled investigation of these two recruitment and administrative strategies is needed to determine the best assessment strategy for studying TTM.

Both groups of participants suffering from TTM showed higher and comparable levels of distress associated with obsessive thoughts and NJREs than unaffected controls. These findings are somewhat in line with the inclusion of TTM in the Obsessive-Compulsive and Related Disorders category of the DSM-5 (American Psychiatric Association, 2013) and with recent data showing that individuals with TTM endorse similar levels of NJREs than patients with OCD, suggesting that these phenomena may play a key role in OCD-related disorders (Sica et al., 2015).

A cross-cultural comparison of present results substantially confirms previous findings on Italian non-clinical hair pullers (Ghisi et al., 2013) suggesting that the phenomenological features characterizing Italians with TTM resemble those observed in Caucasian-American hair pullers. Minimal differences across cultures and countries were observed also in regard to OCD (e.g., Attiullah et al., 2000; Fontenelle et al., 2004); therefore, cross-cultural invariance might represent a characteristic of OCD-related disorders and is a crucial issue to take into account when planning and implementing therapeutic interventions targeting TTM. Indeed, the psychological interventions most effective in reducing TTM symptoms (e.g., Azrin and Nunn, 1973; Mansueto et al., 1997) have been developed and evaluated mainly in the U.S. (e.g., Mouton and Stanley, 1996; Ninat et al., 2000; van Minnen et al., 2003; Woods et al., 2006b). In light of the phenomenological similarities shared by Italian and American hair pullers, the adaptation and implementation of such cognitive and behavioral interventions in the Italian clinical context, as well as the evaluation of their effectiveness, is possible and necessary.

Some shortcomings of the present study must be noted. The small sample sizes limit the generalizability of these findings. The online recruitment strategy, despite allowing more widespread participation, under-sampled those without Internet capability. Furthermore, online assessment and data collection prevent the opportunity of conducting face-to-face clinical interviews of participants referring TTM symptoms; this does not guarantee that all exclusion criteria have been adequately considered (e.g., presence of psychotic symptoms/impaired insight). Another limitation concerns the measure employed to assess hair pulling phenomenology. The IHPQ has yet to be validated in the Italian population, therefore, the reliability of current findings is questionable. Future studies overcoming these limitations are recommended. Furthermore, since previous evidence suggested that affective correlates play a crucial role in the pulling cycle (both triggering and maintaining the behavior; e.g., Diefenbach et al., 2002; Mansueto et al., 2007; Duke et al., 2009, 2010), an investigation of their involvement also in Italian hair pullers is advocated.

Despite the above-mentioned weaknesses, the current study represents the first attempt to shed light on the phenomenological and psychopathological features of TTM in Italian hair pullers. Importantly, the availability and dissemination of clinical data is crucial for both diagnostic and therapeutic purposes, especially because TTM is scarcely known and treated in Italian clinical practice to date.

## Author Contributions

GB, CS, MG: project design; GB, RF, MG: introduction and discussion writing; SC: participants recruitment and testing; GB, ER: data analysis; GB, SC: methods and results writing; MG: project supervision.

## Conflict of Interest Statement

The authors declare that the research was conducted in the absence of any commercial or financial relationships that could be construed as a potential conflict of interest.
